# Novel morphologies of poly(allyamine hydrochloride)–methotrexate nanoassemblies for methotrexate delivery

**DOI:** 10.1039/c7ra12862b

**Published:** 2018-02-20

**Authors:** Wei-Yuan Wang, Xiao-Han Ju, Xiu-Fen Zhao, Xiao-Dong Li, Shu-Ping Li, Fu-Gui Song

**Affiliations:** Jiangsu Key Laboratory of Biofunctional Material, College of Chemistry and Material Science, Nanjing Normal University Nanjing 210023 China lishuping@njnu.edu.cn +86-25-83598678 +86-25-83598280; Shandong Bingkun Tengtai Ceramics Technology Co. Ltd. Zibo 255321 China

## Abstract

Poly(allylamine hydrochloride)–methotrexate (PAH–MTX) nanoassemblies with novel morphologies (*i.e.* nanostrips, nanorolls, nanosheets, and nanospheres) were achieved for the first time *via* supramolecular self-assembly directed by the synergistic action of various non-covalent interactions between PAH and MTX molecules in aqueous solution. Herein, MTX acted in a versatile manner as both a morphology-regulating agent and a small molecular hydrophobic anticancer drug. Moreover, different morphologies presented diverse drug release profiles, which may be caused by the distinctive interactions between PAH and MTX molecules. Synergistically non-covalent interactions, including electrostatic interactions, van der Waals forces, and hydrogen bonding, favored easier matrix corrosion and more rapid drug release of non-spherical structures (*i.e.* nanostrips, nanorolls, and nanosheets) through the ligand exchange process. On the other hand, the highly sealed encapsulation mode for hydrophobic MTX molecules made the nanospheres exhibit slower and better controlled release. In addition, *in vitro* bioassay tests showed that nanostrips displayed the most obvious suppression on the viability of cancer cells among other morphologies, especially after a longer duration. The strategy of using small molecular anticancer drugs not as passively delivered cargoes but as effective molecular building blocks, opens up a new way to develop self-delivering drugs for anticancer therapy.

## Abstract 2

Nanospheres exhibited the best controlled drug release and nanostrips displayed the most obvious suppression of A549 cells.

## Graphical abstract

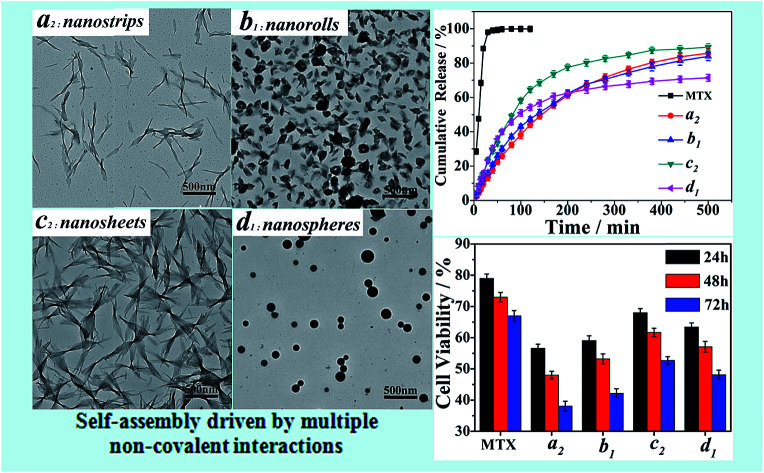

## 1. Introduction

For drug delivery systems, nanoparticle morphology has become a highly attractive research area especially for cancer therapy since it plays critical roles in regulating payload the release, phagocytosis, cell internalization, pharmacokinetics, and bio-distribution of particles.^1^ Tremendous efforts have already been made to explore the morphology effect on drug delivery systems. It has been demonstrated that disc-shaped NPs possessed longer systemic circulation time and higher specific bio-distribution than their spherical counterparts,^2–4^ and that rod-shaped^5,6^ or worm-shaped particles^7,8^ exhibited higher specific and lower nonspecific accumulation than spherical NPs. Moreover, rod-like NPs with higher aspect ratios possessed much faster cell internalization than that of the lower ones.^9^ Since non-spherical NPs presented many impressive advantages, more and more nanocarriers, such as gold NPs,^10^ Magnetic NPs.^11^ silicon dioxide,^12^ and polymeric nanoassemblies,^13^ have been developed with diverse morphologies to evaluate their biological application *in vitro* and *in vivo*.

The nanoassemblies formed by self-assembling multi-structured polymers, especially *via* molecular amphiphiles, have emerged as a kind of helpful and effective system to deliver small molecular drugs owing to their unique properties, such as high loading capacity, increasing solubility of the hydrophobic drugs, long circulation time, stimuli-responsive characteristics, and favorable enhanced permeability and retention (EPR) effect.^1^ Based on that, multiple morphologies of the polymeric nanocarriers, including nanospheres,^2,3,14–17^ nanowires,^3,18^ nanorods,^14^ nano disks,^2,19^ multicompartment micelles,^20^ and vesicles,^3,21–23^ have been developed and presented unexpected virtues during the drug delivery process. As well known, multiple non-covalent interactions have been well engineered to create hierarchical structures, highly complex systems, and functional entities at all scales.^24–26^ Self-assembly system driven by the non-covalent interactions such as van der Waals forces, hydrogen bonding, electrostatic forces, hydrophobic interaction, π–π stacking interaction, and host–guest interaction, may show great potential to create new nanostructures.

**Scheme 1:**
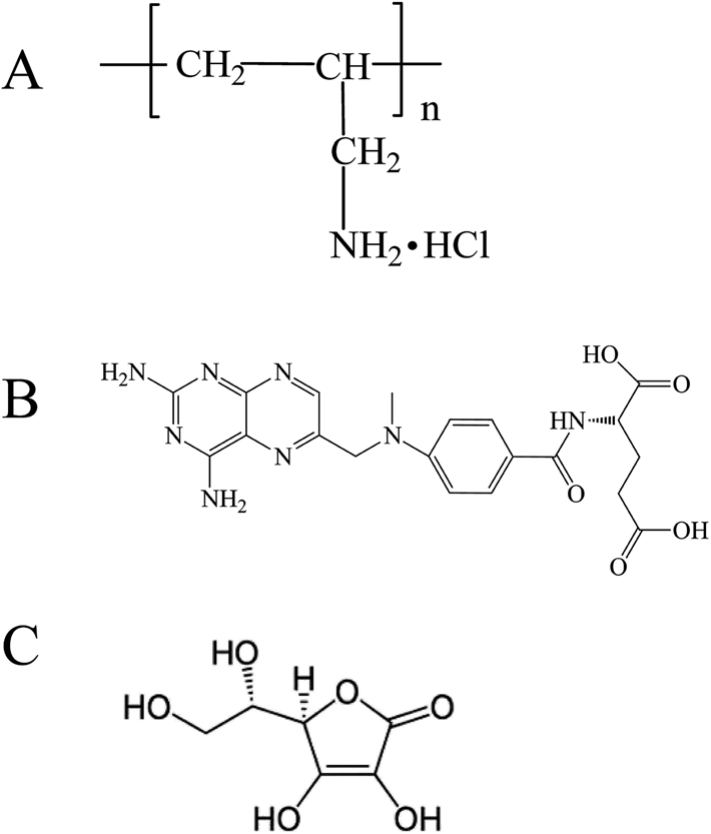
Chemical structures of (A) PAH, (B) MTX, (C) l-AA.

Polyallylamine hydrochloride (PAH, Scheme 1A) possessed abundant primary amine groups and was a cationic, highly water-soluble, and biocompatible polyelectrolyte. Primary amine groups favored Schiff base interaction between PAH and aldehydes (or ketones), resulting in imine groups (–C=N–) and then promoting the formation of diverse nanostructures by molecular self-assembly, such as core–shell PAH–pyrene nanorods,^27^ nanotubes protruding PAH-*graft*-Py microcapsules,^28^ PAH-*g*-porphyrin nanocapsules (*i.e.* microspheres, one-dimensional nanorods (NRs), and wormlike nanostructures (WSs)).^29^ Most typically, PAH was often used to construct multilayers or nanocapsules with poly(sodium 4-styrene sulfonate) (PSS) through the layer-by-layer assembly based on the electrostatic interaction.^30–37^ The resultant PAH nanocapsules also presented biocompatibility, non-toxicity, good encapsulation and controlled release behavior, thus being widely used for gene and drug delivery purposes as well as other biomedical applications. Since many drug molecules were passively encapsulated into the inner side of the pre-assembled nanocapsules, no effort has yet been devoted to construct highly efficient nanomedicines *via* self-assembly directed by the integration of multiple non-covalent interactions between PAH and cargo molecules. The overall balance of these forces may induce interesting and surprising properties for nanostructures. Indeed, the functional primary amine groups and cationic character could open more opportunities for the non-covalent self-assembly between PAH and cargo molecules.

**Scheme 2:**
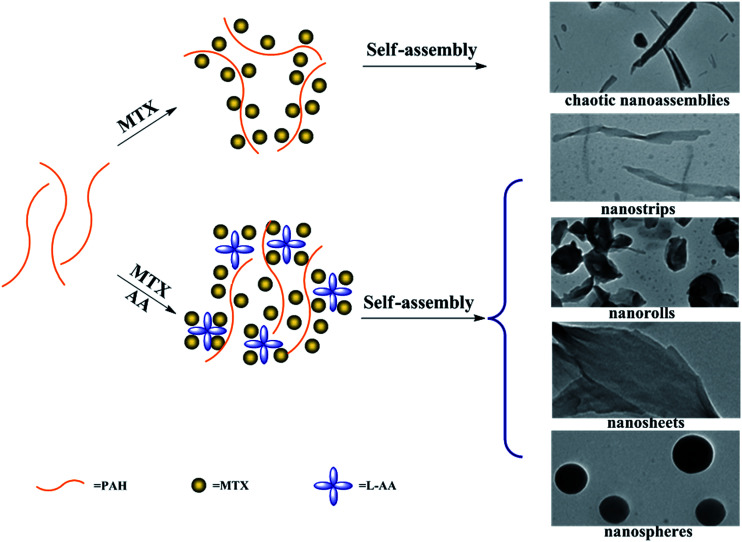
Schematic representation of the morphology changes of PAH–MTX nanoassemblies with and without l-AA.

In the present work, we adopt the supramolecular self-assembly strategy directed by the synergistic action of various non-covalent interactions between PAH and MTX molecules in aqueous solution to develop four kinds of well-defined novel morphologies of poly(allylamine hydrochloride)–methotrexate (PAH–MTX) nanoassemblies (*i.e.* nanostrips, nanorolls, nanosheets, and nanospheres, Scheme 2). MTX (Scheme 1B) here was used efficiently as both a morphology-regulating agent and a small molecular hydrophobic anticancer drug. Moreover, the electrostatic interaction accompanying by the van der Waals forces and hydrogen bonding favored the easier matrix corrosion of non-spherical structures (*i.e.* nanostrips, nanorolls, and nanosheets) through the ligand exchange process and then a rapid drug release while the highly sealed encapsulation mode for hydrophobic MTX molecules made the nanospheres exhibited a slower and better controlled release. Finally, *in vitro* bioassay tests showed that nanostrips displayed the most obvious suppression on the viability of cancer cells than other morphologies, especially after longer duration.

## 2. Experimental section

### 2.1 Materials

Polyallylamine hydrochloride (PAH, weight-average molecular weight 150 000) was supplied by Nitto Boseki Co., Ltd. (Tokyo, Japan). l-Ascorbic acid (l-AA) and ammonia solution (NH_3_·H_2_O) were purchased from Sinopharm Chemical Reagent Co., Ltd. (Shanghai, China). Methotrexate (MTX) was purchased from Huzhou Prospect Pharmaceutical Co. (Zhejiang, CN). Dialysis membrane (molecular weight cut-off = 8000–14 000 Da) was purchased from Shanghai Green Bird Technology development Co., Ltd. Human lung adenocarcinoma cells (A549) were purchased from Shanghai Cell bank (Shanghai, CN). All reagents were of analytical grade and used without further purification. All the aqueous solutions were prepared using Milli-Q water with a resistivity of 18.2 MΩ (Purelab Classic Corp., USA).

### 2.2 Preparation of PAH–MTX nanoassemblies with various MTX contents and temperatures

In a typical synthesis, PAH (0.4 mL, 0.5 mol L^−1^, molarity of PAH given with respect to the repeating unit) was added into 9.6 mL of deionized water under continuous stirring, together with adding l-AA (0.5 mL, 10 mg mL^−1^). Then the mixture (pH 3.5) was treated at 40 °C, 60 °C, 80 °C, respectively. Under each temperature gradient, freshly prepared solutions of MTX/NH_3_·H_2_O (4 mL, pH 7.5) with different concentrations (*i.e.* 3, 4, 5, 6 mg mL^−1^, respectively) were added into the mixture under vigorous stirring for 4 h. After being cooled to room temperature, the corresponding PAH–MTX nanoassemblies were obtained and named as samples a_1_, a_2_, a_3_, b_1_, b_2_, b_3_, c_1_, c_2_, c_3_, d_1_, d_2_, and d_3_. The samples were then stored at 4 °C.

### 2.3 Preparation of PAH–MTX nanoassemblies without l-AA

In a typical synthesis, PAH (0.4 mL, 0.5 mol L^−1^) were added into 9.6 mL of deionized water under continuous stirring at 40 °C. Then freshly prepared solutions of MTX/NH_3_·H_2_O (4 mL, pH 7.5) with different concentrations (*i.e.* 3, 4, 5, 6 mg mL^−1^, respectively) were added into the PAH solution (pH 3.7) under vigorous stirring for 4 h. After being cooled to room temperature, the corresponding PAH–MTX nanoassemblies were obtained and named as samples e_1_, e_2_, e_3_, and e_4_. The samples were then stored at 4 °C.

### 2.4 Preparation of PAH–MTX nanoassemblies with various l-AA contents

In a typical synthesis, PAH (0.4 mL, 0.5 mol L^−1^) were added into 9.6 mL of deionized water under continuous stirring at 40 °C, together with adding l-AA (10 mg mL^−1^) for different amounts (*i.e.* 1.0, 1.5, 2 mL, respectively). The pH values of the resulting mixtures were 3.4, 3.4, 3.3, respectively. Subsequently, freshly prepared solution of MTX/NH_3_·H_2_O (4 mL, 5 mg mL^−1^, pH 7.5) were added into the mixtures under vigorous stirring for 4 h. After being cooled to room temperature, the corresponding PAH–MTX nanoassemblies were obtained and named as samples f_1_, f_2_, and f_3_. The samples were then stored at 4 °C.

### 2.5 Drug loading capacity

The loading capacity of MTX in PAH–MTX nanoassemblies was determined as follows: 0.01 g of PAH–MTX nanoassemblies were dissolved in HCl solution (pH 1.2) and then diluted to 500 mL in volumetric flask. The concentration of MTX was measured by UV-vis spectroscopy at 306 nm. The loading capacity (LC) and encapsulation efficiency (EE) were calculated according to the following equations:
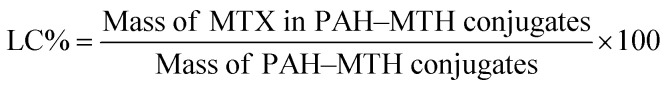

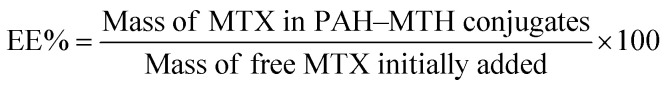


The data were collected in triplicate and are presented in Table 1.

**Table 1:** Characteristic data of PAH–MTX nanoassemblies with different morphologies

Samples	Morphology	Drug loading capacity (%)	Drug entrapment efficiency (%)	Zeta potential (mV)
a_2_	Nanostrip	90.84 ± 0.45	72.67 ± 0.25	+38.3 ± 1.5
b_1_	Nanoroll	88.94 ± 0.33	81.28 ± 0.45	+41.8 ± 2.5
c_2_	Nanosheet	91.08 ± 0.54	76.28 ± 0.35	+43.5 ± 1.6
d_1_	Nanosphere	86.68 ± 0.25	59.81 ± 0.39	+45.5 ± 2.3

### 2.6 *In vitro* drug release

The release profiles of PAH–MTX nanoassemblies were studied by the dialysis with PBS (10 mmol L^−1^, pH 7.4) as the release medium. An appropriate amount of PAH–MTX nanoassemblies were weighed and dispersed in PBS to obtain a final concentration of MTX of 2 mg mL^−1^. Then a fraction of the prepared solution (1 mL of) was added into a dialysis membrane bag (MWCO = 8000–14 000) and immersed in PBS (100 mL). The system was shaken at a speed of 150 rpm at 37 °C. At desired time intervals, 3 mL of sample was withdrawn and replaced with an equal volume of fresh medium. As a control experiment, the release of free MTX from the dialysis bag was measured. All samples were measured by UV-vis spectroscopy at *λ*_max_ = 306 nm. At last, the release profiles were plotted as the relative release percentages of MTX against time. These data were collected in triplicate and are presented in Fig. 4A.

### 2.7 *In vitro* bioassay

*In vitro* bioassays were undertaken with human lung adenocarcinoma cells (A549). Cells were cultured at 37 °C in a humidified atmosphere containing 5% CO_2_ in 75 cm^2^ flasks charged with Dubecco's modified eagle medium (DMEM, 10 mL) and supplemented with 10% (v/v) fetal bovine serum (FBS), penicillin (final concentration of 100 U mL^−1^), and streptomycin (final concentration of 100 mg mL^−1^). When the cells reached 80–90% of cellular confluence, the fault culture cells were differentiated with trypsin–EDTA and then washed twice with freshly prepared PBS (pH 7.4). The cells were then diluted with a volume of DMEM containing 10% (v/v) FBS. For cell proliferation and viability study, cells were seeded onto 96-well plates. The cells were then incubated overnight at 37 °C under CO_2_ atmosphere. After that, the medium in the wells was replaced with fresh medium containing the examined samples (*i.e.* free MTX or PAH–MTX nanoassemblies). The anticancer effect of all samples was examined using the 3-(4,5-dimethylthiazol-2-yl)-2,5-diphenyltetrazolium bromide (MTT) assay at the drug concentration of 100 μg mL^−1^ after 24 h and 48 h of incubation. Simply, the supernatant was removed, followed by the addition of MTT stock solution (5 mg mL^−1^ in PBS, 10 μL, pH 7.4) and DMEM (90 μL) in the absence of FBS into each well. After incubation for 4 h at 37 °C, the supernatant was discarded and DMSO (100 μL) was added into each well. The mixture was shaken before recording the absorbance at 570 nm using a microplate reader (Thermo MK3, USA). Cell viability was calculated using the following formula:^38^cell viability (%) = [(OD_570 (sample)_ − OD_570 (blank)_)/(OD_570 (control)_ − OD_570 (blank)_)] × 100where OD was optical density. The data were collected in triplicate and are presented in Fig. 5.

### 2.8 Characterization

Transmission electron microscope (TEM) images were obtained using a H-7650-HITACHI (Hitachi Medical Co.) machine operating at 200 kV. Samples for TEM were prepared by depositing a drop of sample solution on carbon-coated copper grid and dried at room temperature. Fourier transform infrared spectroscopy (FTIR) spectra were recorded on a Bruker Tensor 27 spectrometer in the wavenumber region of 400 to 4000 cm^−1^ using KBr pellets (with a mass ratio of sample to KBr being 1 : 100), and the resolution of the instrument was 4 cm^−1^. The UV-vis spectra were recorded at room temperature on a UV3600 spectrophotometer (Shimadzu, Japan) equipped with 1.0 cm quartz cells. The X-ray diffraction (XRD) patterns were obtained with a D/max-2500PC rotating anode X-ray powder diffractometer (Rigaku Co.), using Cu Kα radiation (*λ* = 1.5406 Å) from 5° to 45° at a scanning rate of 1° min^−1^. Zeta potential analysis was performed with a PALS Zeta Potential Analyzer, Version 3.43 (Brookhaven Instruments Co.). The morphology change of A549 cells was observed with a light microscope (Olympus CKX41, Japan). The cells were initially seeded into cell culture dishes (35 mm × 10 mm) in DMEM (1 mL) medium with 10% (v/v) FBS. After 24 h for cell attachment, the medium in the wells was replaced with DMEM, free MTX, and sample a_4_ for another 24 h, 48 h or 72 h, and the cells were observed using a light microscope with 10× objective lens.

## 3. Results and discussion

### 3.1 TEM analysis of PAH–MTX nanoassemblies

**Fig. 1:**
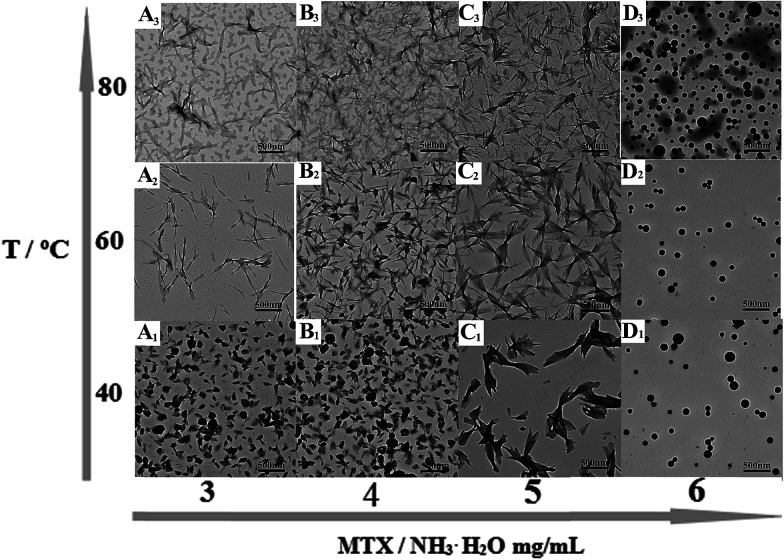
Typical TEM images of PAH-MTX nanoassemblies: (A_1_) sample a_1_, (A_2_) sample a_2_, (A_3_) sample a_3_, (B_1_) sample b_1_, (B_2_) sample b_2_, (B_3_) sample b_3_, (C_1_) sample c_1_, (C_2_) sample c_2_, (C_3_) sample c_3_, (D_1_) sample d_1_, (D_2_) sample d_2_, (D_3_) sample d_3_.

Typical TEM images of the as-prepared PAH–MTX nanoassemblies with various MTX contents and temperatures were shown in Fig. 1. Since multiple non-covalent interactions exhibits between MTX and PAH, such as electrostatic forces, hydrogen bonding, hydrophobic interaction and van der Waals forces, controlling these non-covalent interactions in water is certainly more challenging and dynamical changes in any kind of these interactions may generate distinctive assembly behaviors.^39–41^ As observed, PAH–MTX nanoassemblies at 40 °C changed from the nanorolls (Fig. 1A_1_ and B_1_) to nanosheets (Fig. 1C_1_) and then to nanospheres (Fig. 1D_1_) with increasing MTX contents, indicating that MTX played a critical role in the morphology evolution.^42,43^ Various amounts of MTX were used to tune the grafting density of MTX on PAH chains to form different PAH–MTX polycomplexes, which further assembled into diverse morphologies by the distinct driving forces. When a small amount of MTX was added, synergistic interaction of electrostatic interaction and hydrogen-bonding between MTX and PAH may act as the mainly driving forces to form uniform nanorolls. These nanorolls (Fig. 1A_1_ and B_1_) changed into nanostrips (Fig. 1A_2_ and B_2_) with increasing temperature from 40 °C to 60 °C and corrosion phenomenon happened at 80 °C (Fig. 1A_3_ and B_3_). The phenomena may be due to the fact that increasing temperature could greatly hinder the formation of hydrogen-bonding and destruct the original balance of synergistic non-covalent interactions at 40 °C, therefore resulting in a temperature-induced aggregation and concomitant changes of the aggregate structures.^44,45^ When a increasing amount of MTX was added, nanosheets were formed, which became more regular and uniform at 60 °C (Fig. 1C_2_) than those at 40 °C (Fig. 1C_1_) and changed into narrower nanostrips (Fig. 1C_3_) at 80 °C. Obviously, temperature has a small effect on the morphology changes with the increase of MTX, which may be explained by the idea that increasing MTX lead to the enhancement of intermolecular non-covalent interactions among PAH–MTX polycomplexes, which can greatly compensate for the lost hydrogen-bonding that are destroyed by higher temperatures.^46,47^ However, when an excess amount of MTX was added, nanospheres were formed (Fig. 1D_1_) and increasing temperature can not affect the morphology at all, indicating that stronger hydrophobic interaction may prevail to play a much crucial role in the assembly process.^39^ Generally, all the observations suggested that MTX and temperature cooperatively impacted on the morphology changes at a certain degree.

**Fig. 2:**
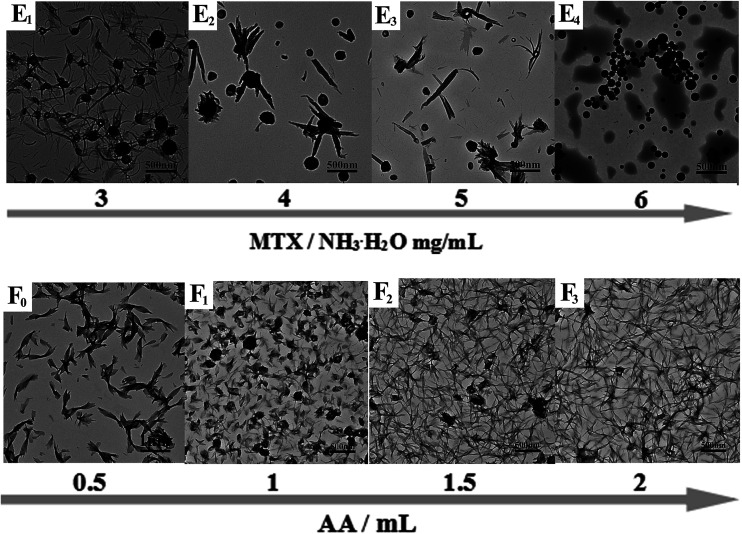
Typical TEM images of PAH–MTX nanoassemblies: (E_1_) sample e_1_, (E_2_) sample e_2_, (E_3_) sample e_3_, (E_4_) sample e_4_, (F_0_) sample c_1_, (F_1_) sample f_1_, (F_2_) sample f_2_, (F_3_) sample f_3_.

Typical TEM images of the as-prepared PAH–MTX nanoassemblies involving various l-AA contents were shown in Fig. 2. As observed, almost all the PAH–MTX nanoassemblies (Fig. 2E_1_–E_3_) exhibited chaotic and irregular morphology without adding any amount of l-AA. Sample e_4_ (Fig. 2E_4_) was nanospheres but with extremely irregular particle diameters. Owever, when introducing a certain amount of l-AA, PAH–MTX nanoassemblies became even regular and uniform. When the concentration of MTX was 5 mg mL^−1^, PAH–MTX nanoassemblies evolved from nanosheets (Fig. 2F_0_) to nanorolls (Fig. 2F_1_), and finally to nanostrips (Fig. 2F_2_ and F_3_) with increasing l-AA. In addition, the pH values of PAH solutions with adding different amounts of l-AA (0, 0.5, 1, 1.5, and 2 mL, respectively) were 3.7, 3.5, 3.4, 3.4, and 3.3, respectively, indicating very small pH changes of the initial reaction solutions. Hence, the possibility could be ruled out that various pH induced different assembly behaviors.^43^ Considering many active hydrogen-bonding sites characterizing l-AA (Scheme 1C), such as four hydroxyls and a lactone, the phenomenon of morphology changes may be explained by the idea that l-AA additionally introduce abundant intermolecular hydrogen-bonding, which can enhance the intermolecular interactions, guide the assembly behaviors and improve the stability of the self-assembly structures.^41,46–52^

### 3.2 FTIR, UV-vis and XRD analysis of PAH–MTX nanoassemblies

**Fig. 3:**
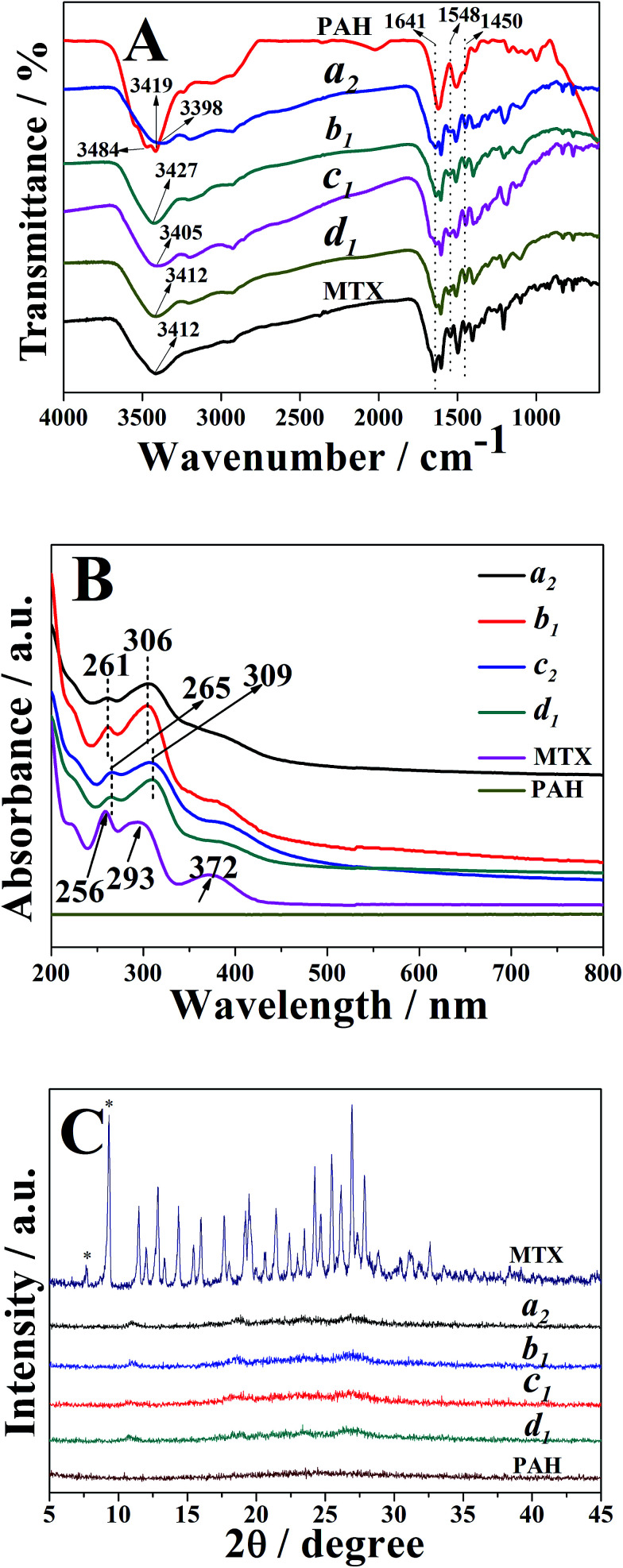
(A) FTIR spectra of free MTX, PAH, samples a_2_, b_1_, c_2_ and d_1_. (B) UV-vis absorption spectra of free MTX, PAH, samples a_2_, b_1_, c_2_ and d_1_. (C) XRD patterns of free MTX, PAH, samples a_2_, b_1_, c_2_ and d_1_.

The FTIR spectra of the typical nanostrips, nanorolls, nanosheets and nanospheres (*i.e.* samples a_2_, b_1_, c_2_ and d_1_, respectively) were illustrated in Fig. 3A. As observed, all samples exhibited peaks at 1641 cm^−1^, 1548 cm^−1^ and 1450 cm^−1^, respectively, which were assigned to the stretching vibration of the C=O group, amide II N–H bending mode and carbon–carbon stretching vibrations in benzene ring of MTX, respectively.^53^ These observations indicated the successful loading of MTX into PAH–MTX nanoassemblies. As for PAH, two strong broad absorption peaks at 3484 cm^−1^ and 3419 cm^−1^ corresponded to the N–H asymmetric and symmetric stretching modes of the primary amine groups. As for PAH–MTX nanoassemblies, disappearance of the corresponding double peaks and appearance of single peak at the relatively low wave number between 3398 and 3427 cm^−1^, pointed out the strong hydrogen-bonding or ionic interaction between –NH_2_ groups and other polar groups.^53–55^ This observation may reveal the occurrence of molecular assembly and then the subsequent formation of different nanoassemblies.

As for UV-vis measurements (Fig. 3B), pure MTX in alkaline solution (pH 7.5) exhibited two obviously characteristic absorption peaks at 256 nm and 293 nm, corresponding to π → π* (carboxylate) and *n* → π* (enone) transitions, respectively.^56,57^ Obviously, PAH presented no characteristic absorption from 200 nm to 800 nm. As for the PAH–MTX nanoassemblies, samples a_2_ and b_1_ exhibited two distinct peaks both at 261 nm and 306 nm, presenting a small red shift of 5 nm and 13 nm. Meanwhile, samples c_2_ and d_1_ exhibited two distinct peaks both at 265 nm and 309 nm, presenting a small red shift of 9 nm and 16 nm, respectively. These different red-shifts may be caused by the synergistic non-covalent interactions, including electrostatic interactions, hydrogen bonding, hydrophobic interaction, and van der Waals forces.^56^

Generally, drug distribution into particles was directly correlated with drug release rate and bioavailability, therefore being an important parameter in the development of nanomedicine. The XRD patterns of free MTX, PAH, samples a_2_, b_1_, c_2_ and d_1_ were shown in Fig. 3C. As observed, free MTX occurred in a crystalline state, which was attributed to the well-defined diffraction peaks at 2*θ* of 7.6 and 9.2, characteristic of its trihydrate form.^58,59^ It was well-known that the width of X-ray diffraction peak was related to the size of crystallite and the broadened peak usually results from imperfect crystal. Hence, the broad peak of PAH indicated a amorphous phase. Compared to free MTX and PAH, however, almost no crystallinity was observed from different PAH–MTX nanoassemblies, indicating a fully molecular dispersion of MTX into the polymeric matrix or amorphization during drying.^59,60^

### 3.3 Drug loading capacity and *in vitro* release analysis of PAH–MTX nanoassemblies

Drug loading capacities of all samples were listed in Table 1. As observed, the loading capacities of samples a_2_, b_1_, c_2_ and d_1_ were 90.84 ± 0.45, 88.94 ± 0.33, 91.08 ± 0.54, and 86.68 ± 0.25, respectively. Moreover, their encapsulation efficiencies were 72.67 ± 0.25, 81.28 ± 0.45, 76.28 ± 0.35, and 59.81 ± 0.39, respectively. The data showed that different PAH–MTX nanoassemblies presented high and approximate loading capacities but distinct encapsulation efficiencies. Compared with other shapes, the nanospheres exhibited the lowest encapsulation efficiency, indicating that increasing MTX did not evidently enhance drug loading capacity but apparently regulate the morphology evolution.^42,43^ In addition, highly positive zeta potentials suggested that hydrophilic amino groups exposed on the surface of the nanoassemblies.

**Fig. 4:**
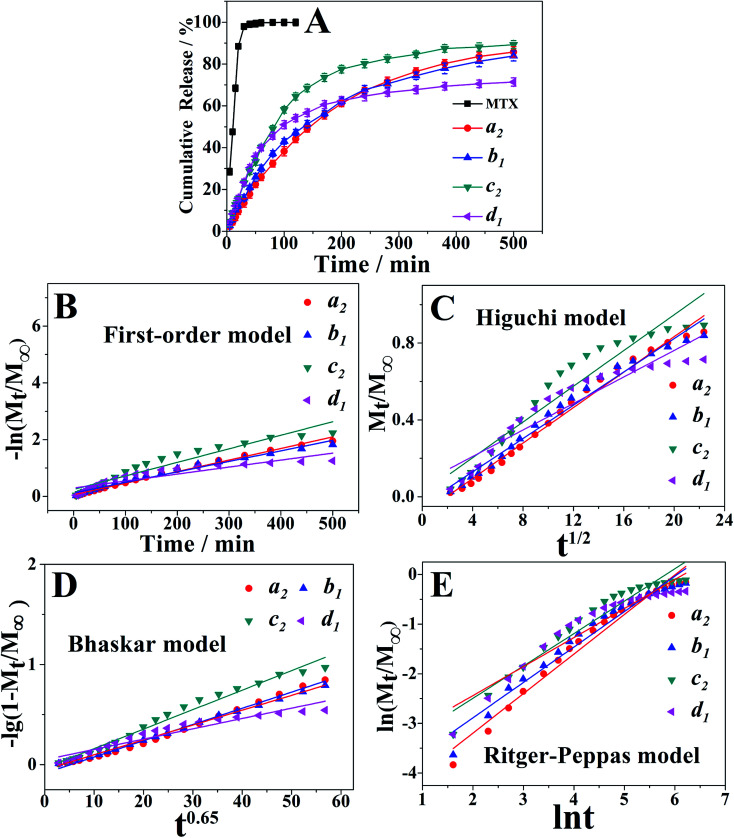
(A) Release profiles of free MTX, samples a_2_, b_1_, c_2_ and d_1_. (B–E) Plots of different kinetic models for the release of MTX from the PAH–MTX nanoassemblies.

Meanwhile, typical *in vitro* release profiles of MTX from samples a_2_, b_1_, c_2_ and d_1_ were investigated and illustrated in the Fig. 4A. The control experiment showed a complete MTX diffusion across the dialysis membrane within 120 min. With a rapid release of MTX at the initial stage and a relatively slower one at the latter stage, PAH–MTX nanoassemblies, however, exhibited a sustained drug release effect. Such a two-step and sustained release behavior could maintain the drug level within a therapeutic window.^61^ Notably, different morphologies of nanoassemblies presented diverse release profiles,^62,63^ which may be due to the different interactions between PAH and MTX. Release rates of the four samples within 200 min were 61.11%, 62.26%, 77.55%, and 62.46%, respectively. Within 500 min, the release rates reached to 85.77%, 83.56%, 89.27%, and 71.41%, respectively. As well known, PAH was a positively charged polyelectrolyte with strong hydrophilicity while MTX was a hydrophobic drug but possessed negative charge in basic solution. As for sample d_1_, MTX molecules as hydrophobic parts were mainly encapsulated into the inner side while the hydrophilic amino groups of PAH exposed outside. The highly sealed and spherical structure caused a finally lower rate of matrix erosion and then a slower drug diffusion.^64^ The initially fast release rate may be due to the fact that many MTX molecules also physically adsorbed on the surface of nanospheres based on the electrostatic interaction. As for samples a_2_, b_1_, c_2_, positively charged PAH chains and negatively charged MTX molecules cross-linked into regular nanoassemblies based on the mainly electrostatic self-assembly as well as other non-covalent interactions (*i.e.* van der Waals forces and hydrogen bonding). Accordingly, it was more favorable for the exchange process of negative ligands to induce matrix erosion of PAH–MTX nanoassemblies and then a fast drug diffusion.^65^ Consequently, these samples presented higher release rates after 500 min.

The drug release based on the PAH–MTX nanoassemblies could be controlled by any of the following steps: (1) dissolution of PAH–MTX nanoassemblies; (2) ion-exchange process between MTX-containing nanoassemblies and the phosphate anions in buffer solution.^66^ To gain more insights into the mechanism of drug release, four equations were often applied:^67^ the first-order equation (eqn (1)), the Higuchi equation (eqn (2)), the Bhaskar equation (eqn (3)), the Ritger–Peppas (eqn (4)), see the following:

1 ln(1 − *M*_*t*_/*M*_∞_) = −*k*_1_*t*

2 *M*_*t*_/*M*_∞_ = *k*_H_*t*^1/2^

3 ln(1 − *M*_*t*_/*M*_∞_) = −*k*_B_*t*^0.65^

4 *M*_*t*_/*M*_∞_ = *kt*^*n*^

in above equations, *M*_*t*_/*M*_∞_, *t*, *k* were the fractional drug release, release time and the corresponding release rate constant, respectively.

On the basis of the four different kinetic models, the fitting results of drug release profiles were given in Fig. 4B–E. Furthermore, the corresponding linear correlation coefficients (*R*) and *n* values obtained from the fittings were summarized in Table 2. As can be seen, Bhaskar model and R–P model gave more reasonable fitting coefficients of *R* = 0.95–0.99, indicating that these two models can better describe the release mechanism. For all samples, Bhaskar equation fitted the release data best, indicating that the release process mainly belonged to intraparticle-controlled diffusion. Additionally, the R–P equation were used to explain drug diffusion and dissolution of polymeric matrix. The value of *n* < 0.45 corresponded to the drug diffusion control, which was based on ion exchange process; *n* > 0.89 was attributed to the dissolution of PAH–MTX nanoassemblies; 0.45 < *n* < 0.89 was due to the cooperation of PAH–MTX nanoassemblies dissolution and drug diffusion. The release process of all samples with release value of *n* between 0.5842 and 0.7969 (0.45 < *n* < 0.89) belonged to the combination behavior control, which can be based on the fact that the MTX anions diffused from the inner side of PAH–MTX nanoassemblies to the surface of particles accompanying the matrix corrosion of the nanoassemblies.

**Table 2:** Fitting parameters of different kinetic models for the release of MTX from the PAH-MTX nanoassemblies

Samples	First-order model	Higuchi model	Bhaskar model	Ritger–Peppas model	
	*R*	*R*	*R*	*R*	*n*
a_2_	0.9958	0.9928	0.9967	0.9868	0.7969
b_1_	0.9897	0.9889	0.9993	0.9804	0.7130
c_2_	0.9623	0.9575	0.9902	0.9692	0.6523
d_2_	0.9058	0.9386	0.9595	0.9508	0.5842

### 3.4 Cell viability test of PAH–MTX nanoassemblies

**Fig. 5:**
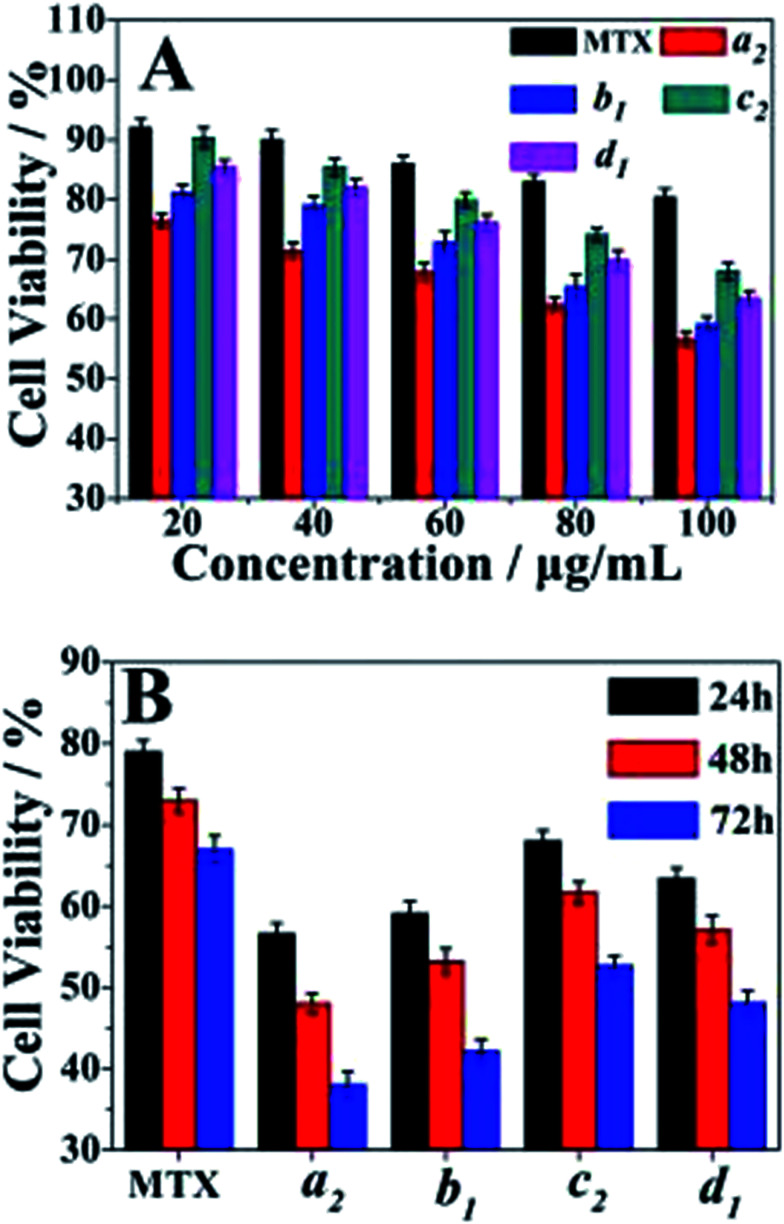
(A) Comparison of cell viabilities for free MTX, samples a_2_, b_1_, c_2_ and d_1_ at various concentrations after 24 h of incubation; (B) comparison of cell viabilities for free MTX, sample a_2_, b_1_, c_2_ and d_1_ after different incubation time at the concentration of 100 μg mL^−1^. All cell viability data were obtained from three separated experiments and error bars represent standard error (*n* = 3).

Cell cytotoxicity of the PAH–MTX nanoassemblies was evaluated by the bioassay test using A549 cells and was presented in Fig. 5. The Fig. 5A showed the cell viabilities of free MTX, samples a_2_, b_1_, c_2_ and d_1_ measured at different concentrations after 24 h of incubation. The data revealed that samples a_2_, b_1_, c_2_ and d_1_ presented obvious suppression efficiency on the A549 cells when the concentration of PAH–MTX nanoassemblies increased while the cell viability of the samples treated with free MTX remained almost the same. Thus, the PAH–MTX nanoassemblies were more efficient than free MTX in the suppression of the cancer cells. Two factors could account for the better suppression. One was that the electrostatic attraction between positively charged PAH–MTX nanoassembelies and negatively charged cell membrane were more favorable for cell adhesion and internalization towards the nanoassembelies.^30,68,69^ The other may be due to the receptor-mediated endocytosis of PAH–MTX nanoassemblies, where Janus-like MTX acted as an early-phase targeting ligand coordinated to a late-phase anticancer drug.^14^ Notably, under the same drug concentration, different morphologies of PAH–MTX nanoassemblies exhibited differences in cell viabilities. Relatively, nanostrips presented strongest suppression towards the cancer cells while nanosheets showed the weakest. This may be due to the fact that strip structure was more favorable for the cell endocytosis compared with the nanosheets.^9,70^ The Fig. 5B showed the time dependence of the cell viabilities of PAH–MTX nanoassemblies at the concentration of 100 μg mL^−1^. The cell viability of all samples constantly decreased as the incubation time increased, suggesting a controlled and sustained drug release process.

**Fig. 6:**
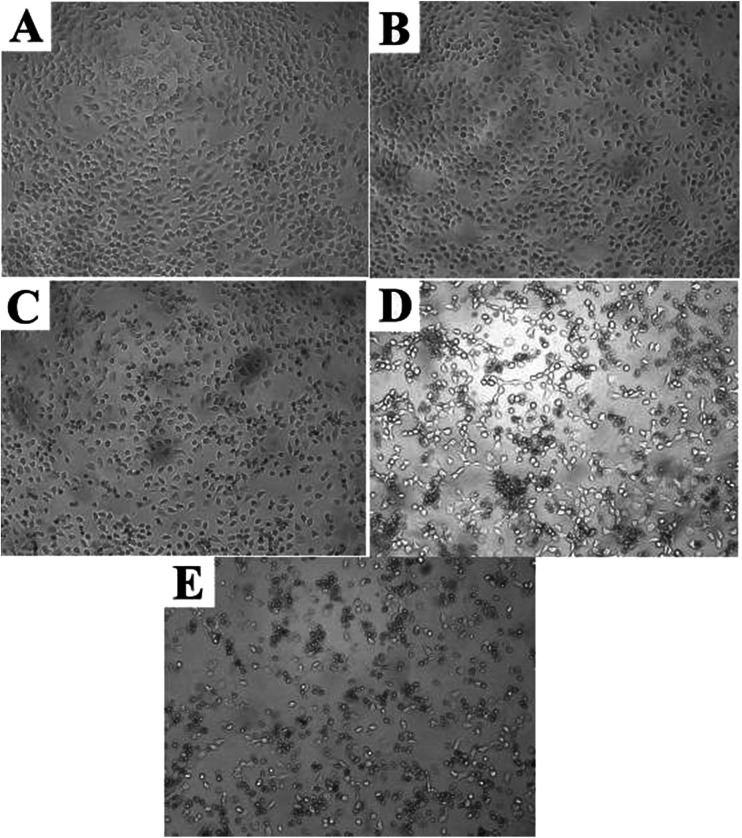
Morphology changes of A549 cells treated with various samples at the concentration of 100 μg mL^−1^: (A) DMEM for 24 h; (B) free MTX for 24 h; (C) sample a_2_ for 24 h; (D) sample a_2_ for 48 h; (E) sample a_2_ for 72 h.

Complementary to the MTT assay, morphology changes of the A549 cells treated with DMEM, free MTX and sample a_2_ were displayed in Fig. 6. Compared with the reference morphology of the A549 cells treated with DMEM (Fig. 6A), the cell form of the samples treated with free MTX (Fig. 6B) slightly changed, indicating that the uptake of free MTX was limited. However, the cell form obviously changed after treated with sample a_2_ (Fig. 6C), and the co-existence of spindle and shrinkable cells meant that part of A549 cells were withered or close to death after incubation of 24 h. After 48 h, more cells (Fig. 6D) displayed circular forms, revealing that the cells suffered from apoptosis. After 72 h, the cells state (Fig. 6E) was even in worse, revealed by the corrosion of almost all the cells. These observations suggested that PAH–MTX nanoassemblies were promising candidates to deliver MTX for anticancer therapy.

## 4. Conclusions

In summary, we have successfully developed four kinds of well-defined novel morphologies of PAH–MTX nanoassemblies (*i.e.* nanostrips, nanorolls, nanosheets, and nanospheres) *via* supramolecular self-assembly strategy directed by the synergistic action of multiple non-covalent interactions between PAH and MTX molecules in aqueous solution. Herein, MTX acted versatilely as both a morphology-regulating agent and a small molecular hydrophobic anticancer drug. Moreover, non-spherical structures (*i.e.* nanostrips, nanorolls, and nanosheets) favored easier matrix corrosion and then a rapid drug release through the ligand exchange process while the nanospheres exhibited a slower and better controlled release due to the highly sealed encapsulation mode for hydrophobic MTX molecules. In addition, the nanostrips displayed the most obvious suppression on the cancer cells among all the morphologies, especially after longer duration. Finally, this strategy can be extended to construct nanostructures of other types of anticancer drugs and thus presents new opportunities for the development of self-delivering drugs for anticancer therapy.

## Conflicts of interest

There are no conflicts to declare.

## Supplementary Material
